# Outcomes and Complications of Implant-Based Breast Reconstruction in Patients With Previous Cosmetic Augmentation: A Systematic Review

**DOI:** 10.1093/asjof/ojaf049

**Published:** 2025-05-26

**Authors:** Virginia H R Monteil, Edward T C Dong, Carlo M Oranges

## Abstract

Breast augmentation is a very popular cosmetic surgery—which may subsequently influence the technique and outcomes of breast reconstruction in the event of breast cancer. After mastectomy, several surgical approaches are available for this patient population. The authors of this study aim to systematically review the available methods, their associated complications, and patient satisfaction outcomes. A comprehensive search of the PubMed/MEDLINE database was conducted by structured search algorithms to identify relevant studies. Three reconstruction techniques were categorized, and related complications and outcomes were compiled into tables for analysis. A total of 11 studies on implant-based breast reconstruction met the inclusion criteria. Three main surgical techniques were identified: tissue expander-to-implant (TE; *n* = 250 breasts) reconstructions, implant-sparing mastectomies (ISMs; *n* = 196 breasts), and direct-to-implant (DTI; *n* = 137 breasts) reconstructions. The overall complication rates were 54.8% for TE, 22.4% for ISM, and 25.5% for DTI, and the most frequent complications were infection, necrosis, and capsular contraction, respectively. Patient satisfaction in TE and DTI groups ranged from average to high for the 3 domains explored, namely psychosocial well-being, breast satisfaction, and sexual well-being (ISM not reported). Three primary techniques to address implant-based breast reconstruction in patients with previous breast augmentation are described in literature: TE, ISM, and DTI. Patients necessitating TE present a high complication rate, whereas ISM and DTI show a safer profile. Patient satisfaction evaluated with BREAST-Q scores in TE and DTI groups appeared to vary from average to high.

**Level of Evidence: 3 (Therapeutic):**

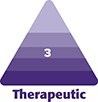

Breast augmentation has been one of the most popular cosmetic surgeries in the United States since the FDA approval of silicone implants in 2006, with 249,560 procedures reported by the Cosmetic Data Bank in 2023.^1,2^ Globally, it remains the second most performed cosmetic procedure, representing 12% of all surgeries.^3^ This intervention typically involves the placement of saline or silicone implants, either submuscularly or subglandularly.^4-8^ Fat grafting has also emerged as a viable autologous alternative and can also be used for corrections.^9-12^

Concurrently, breast cancer remains the most frequent cancer among women, with over 2 million new cases reported in 2022.^13^ Different lines of treatment exist, including surgical (by mastectomy or lumpectomy), radiotherapy, chemotherapy, and immunotherapy.^14-20^ In case of mastectomy, several reconstructive options are available, of which 80% are implant based.^21-25^ Since Cronin and Gerow introduced silicone gel breast implants in 1963, new reconstructive possibilities have emerged. This includes the development of autologous flaps in the 1970s and Radovan's tissue expanders for implant reconstruction in 1982.^26-32^ However, in previously augmented patients, if no indications for removal exist, an alternative technique—implant-sparing mastectomies (ISMs)—may be performed.^33,34^

Breast reconstruction after mastectomy in patients with previous breast augmentation poses unique challenges because of altered anatomy and compromised tissue quality.^10^ Complications such as infection, tissue necrosis, and implant loss are specific concerns, alongside the critical factor of patient satisfaction.^10^ Reconstructive approaches include 1-stage techniques, such as direct-to-implant (DTI) reconstruction and ISM, as well as a 2-stage approach involving tissue expander-to-implant (TE) reconstruction.

TE is typically preferred in cases with compromised skin flaps, when significant skin is removed during mastectomy or when radiation therapy is anticipated, potentially reflecting a greater complexity of the case or procedure.^35,36^ TE was the most performed implant-based reconstruction in the United States in 2023, according to American Society of Plastic Surgeons (ASPS), followed by DTI, albeit having a longer and more painful treatment duration.^37,38^ In contrast, DTI breast reconstruction has been gaining popularity as an alternative with a single surgery and good immediate cosmetic outcomes.^39,40^

The current literature describes several key benefits of ISM compared with TE because it reduces the number of follow-up visits, making it more convenient and cost-effective for patients.^41^ Nonetheless, counter indications frequently appear; Robbins et al reported that 98.9% of patients required implant removal during reconstruction. This may be because of aged, ruptured or leaking implants, site infection, implant exposure, capsular contracture, or tumor proximity to implants. Additionally, ISM is rarely possible in practice in case of subglandular implant position.^33,38^

For women who have had previous cosmetic augmentation, prepectoral (subglandular) reconstruction has become an increasingly preferred option for patients with thicker mastectomy flaps. This technique provides adequate implant coverage while avoiding muscle disruption; which may still be required for a submuscular approach in patients with thinner mastectomy flaps.

To avoid the need for additional materials like acellular dermal matrix (ADM), the existing implant capsule may also be used and adapted to provide a natural covering for the new implant. This form of capsular preservation through controlled capsulotomies is a good option in patients with previous submuscular implants.^35,41^ However, the existing capsule may also increase capsular contracture rates after placement of new implants.^35^

Given the increasing prevalence of breast augmentation and breast cancer, it is of utmost importance to understand reconstruction in these cases. However, the literature on this topic remains sparse, and there is no comprehensive analysis of the existing evidence or consensus to guide patients and surgeons in their choice of technique.

This systematic review aims to assess the complication rates and patient satisfaction associated with the primary surgical approaches for implant-based breast reconstruction in patients with a history of previous augmentation.

## METHODS

The study protocol was preemptively registered on PROSPERO (registration ID: 635468), and the Preferred Reporting Items for Systematic Reviews and Meta-Analyses guidelines were followed in this review.

### Search Strategy

Our preliminary screening revealed that the existing literature describes 3 implant-based techniques for this topic: TE reconstruction, ISM, and DTI reconstruction. The definitive literature search was conducted on PubMed/MEDLINE on January 13, 2025, by utilizing a combination of keywords, including “breast augmentation” or “augmented breasts” or “cosmetic breast surgery” and “breast reconstruction,” along with synonyms for these 3 methods: “direct-to-implant” or “tissue expander,” as well as “implant-sparing mastectomy,” and relevant MeSH terms. Details of the search queries are provided in Supplemental Table 1. No restrictions on publication dates or languages were applied (Supplemental Table 1).

### Eligibility Criteria

We included studies that provide a comprehensive overview of reconstructive outcomes and complications in patients who underwent mastectomy with previous breast augmentation or augmentation auxiliary procedures for TE, ISM, and DTI reconstructions. Reviews about breast conservation therapy or autologous reconstruction were excluded.

### Article Selection

Selection criteria for the systematic review were established by the Population, Intervention, Comparison, Outcomes and Study principles. Primary outcomes included postoperative complications such as necrosis, hematoma, seroma, infection, explantation, wound complications, and capsular contracture, along with the rate of readmission to the operating room. The secondary outcome evaluated was patient satisfaction measured by utilizing BREAST-Q scores. Articles identified through the search query were initially processed by 2 authors (V.H.R.M. and E.T.C.D.), by utilizing the Rayyan web-app (Rayyan, Cambridge, MA) accessed January 15, 2025). Titles and abstracts were reviewed during the prescreening phase, duplicates were removed, and full-text reviews were conducted for eligible articles. The senior author (C.M.O.) supervised these steps to ensure the accuracy of inclusion decisions and helped solve any discrepancies (Supplemental Table 2).

### Data Extraction and Analysis

Selected articles underwent a detailed review. Data extracted included study design and population characteristics, as well as our predefined primary and secondary outcomes, and was systematically integrated into a standardized Excel spreadsheet (Version 16.83, Microsoft Corp., Redmond, WA) by the first and the second authors (V.H.R.M. and E.T.C.D.), under the supervision of the senior author (C.M.O.). Descriptive statistics were realized by utilizing Excel.

## RESULTS

**Figure 1.**
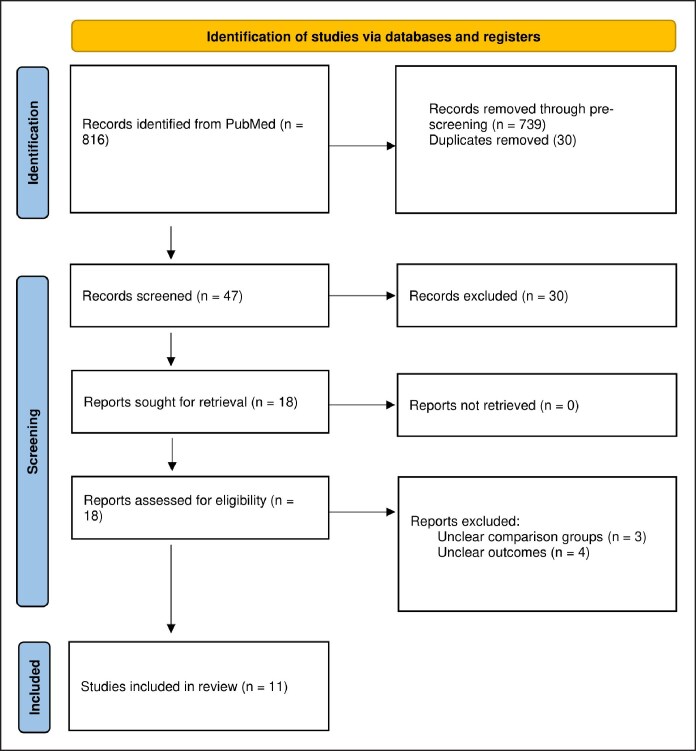
Preferred Reporting Items for Systematic Reviews and Meta-Analysis flowchart of the article selection process. The flowchart details the article identification, screening, and inclusion process.

A total of 816 publications were initially identified through the PubMed/MEDLINE search for potential inclusion. After reviewing titles and abstracts, 739 were excluded because of ineligibility, and 30 duplicates were removed, leaving 47 articles for further screening based on their relevance. During the study selection process, the references of the included articles were also examined, resulting in the identification of 1 additional article, leading to a final selection of 11 articles (Figure 1).

### Study and Patient Characteristics

The studies were published between 2012 and 2024. They included 3 prospective studies and 8 retrospective studies: 7 conducted in the United States, 3 in Italy, and 1 in the United Kingdom. Together, these studies represented a total of 419 patients (601 breasts), with 583 breasts serving for complication rate calculations and 213 patients (282 breasts) for BREAST-Q score ranges. Across the selected studies, mean age range was comprised between 45 and 55.7 years old, and mean BMI ranged from 21.3 to 24.8 kg/m^2^. Thirty-eight patients also underwent radiation therapy. Unfortunately, the number of breasts was not specified for this group. Details about all studies are presented in Table 1. Mean follow-up duration ranged from 15.3 months to 3 years. All complication rates are presented on a per breast basis in Table 2. According to our predefined groups, we categorized 250 breasts in TE group, 196 breasts in ISM group, and 137 breasts in DTI group (Tables 1, 2).

**Table 1.** Summary of Studies on Breast Augmentation

Authors (year)	Study design	Level of evidence	Total no. of breasts (patients)	Geographic location	Mean age, years	Technique (no. of breasts)	Complications
Salgarello et al (2024)^39^	Retrospective review	3	50 (38)	Italy	54	DTI = 50	Infection (*n* = 2), delayed wound closure (*n* = 1), capsular contracture (*n* = 3), implant dislocation (*n* = 1), contour deformity (*n* = 3), animation deformity (*n* = 2)
Tedeschi et al (2024)^10^	Prospective review	—	47 (47)	Italy	55.7	DTI = 21 TE = 26	Necrosis (*n* = 4), seroma (*n* = 3), explantation (*n* = 3), delayed wound closure (*n* = 2)
Prabhakaran et al (2016)^38^	Retrospective review	—	63 (53)	USA	51.19	TE = 18 ISM = 45	Not specified (*n* = 11)
Sbitany et al (2014)^42^	Prospective review	2	51 (34)	USA	45.4	TE = 51	Necrosis (*n* = 3), hematoma (*n* = 1), seroma (*n* = 6), infection (*n* = 17), explantation (*n* = 5), delayed wound closure (*n* = 2), prosthesis exposure (*n* = 1)
Burke et al (2020)^43^	Retrospective review	—	121 (73)	USA	48.4	ISM = 121	Necrosis (*n* = 10), hematoma (*n* = 4), seroma (*n* = 1), infection (*n* = 1), explantation (*n* = 3), delayed wound closure (*n* = 3), unspecified return-to-OR (*n* = 12)
Le et al (2021)^41^	Retrospective review	—	30 (15)	USA	47	ISM = 30	Necrosis (*n* = 3), seroma (*n* = 1), epidermiolysis (*n* = 2)
Roostaeian et al (2015)^35^	Retrospective review	—	63 (11)	USA	47	DTI = 19 TE = 44	Hematoma (*n* = 2), infection (*n* = 2), explantation (*n* = 1), delayed wound closure (*n* = 3), capsular contracture (*n* = 9)
Liu et al (2024)^44^	Retrospective review	4	111 (67)	USA	52	TE = 111	Hematoma (*n* = 5), seroma (*n* = 12), infection (*n* = 18), delayed wound closure (*n* = 25), unspecified return-to-OR (*n* = 22)
Elliott et al (2014)^45^	Retrospective review	—	35 (20)	USA	45.1	DTI = 35	Hematoma (*n* = 1), infection (*n* = 3), explantation (*n* = 1)
Baker et al (2022)^46^	Prospective review	—	62 (49)	UK	45	DTI or TE No of breasts was not reported per group	Not reported per group
Salgarello et al (2012)^47^	Retrospective review	—	12 (12)	Italy	45	DTI = 12	Seroma (*n* = 1)

DTI, direct-to-implant; ISM, implant-sparing mastectomies; TE, tissue expander-to-implant.

**Table 2.** Summary of Complications for Each Technique Group (Data Reported on a Per Breast Basis, *n* = 583 Breasts)

Complication	TE group (*n* = 250)	%	ISM group (*n* = 196)	%	DTI group (*n* = 137)	%
Capsular contraction	5	2.0	0	0	7	5.11
Delayed wound closure	28	11.2	3	1.53	5	3.65
Explantation	6	2.4	3	1.53	5	3.65
Hematoma	7	2.8	4	2.04	2	1.46
Infection	37	14.8	1	0.51	5	3.65
Necrosis	5	2.0	13	6.63	2	1.46
Complete	2	0.8	—	—	2	1.46
Partial	1	0.4	—	—	0	0
Nipple	2	0.8	—	—	0	0
Return-to-OR (not otherwise specified reasons)	22	8.8	12	6.12	—	—
Seroma	19	7.6	2	1.02	3	2.19
Other	8	3.2	6	3.0	6	4.38
Total complications	137	54.8	44	22.4	35	25.55

DTI, direct-to-implant; ISM, implant-sparing mastectomies; TE, tissue expander-to-implant.

### Primary Outcomes

The TE group consisted of 5 studies and exhibited the highest overall complication rate of 54.8% and a return-to-OR rate of 8.8%, although specific reasons for reoperation were not provided. The most common complications in this group were infection (14.8%) and delayed wound closure (11.2%).

Three studies treated ISM, resulting in an overall complication rate of 22.4% with necrosis noted as the most common complication (6.63%). The return-to-OR rate for this group was slightly lower than for TE, at 6.12%, but reasons were also generally unspecified. Other complications were much less frequent (<3%).

It should be noted that in the study by Prabhakaran et al, only the total number of complications per group (TE and ISM) was provided, without further details. In this case, these totals were included in Table 2 under the “Other” section.^38^

Finally, we identified 5 articles on DTI, with an overall complication rate of 25.55%. Two of these studies further compared subglandular to submuscular placement. Salgarello et al noted a slight increase in the incidence of complications and aesthetic dissatisfaction in the submuscular group (10 vs 8), even if this difference is statistically insignificant.^39^ In this group, the most frequent complication was capsular contraction (5.11%), and the least frequent were necrosis and hematoma (both 1.46%). The “other” category of complications in the DTI group consisted entirely of late complications (implant dislocation, contour deformity, and animation deformity). The data were taken from Salgarello et al and may be explained by the significantly longer follow-up period of 3 years.

### Secondary Outcomes

The secondary outcome assessed in this review was BREAST-Q scores, which provided insights into patient-reported outcomes regarding quality of life and satisfaction. Five studies contributed data for this category (4 for DTI, 3 for TE); however, no data was available to analyze the BREAST-Q results for the ISM group. In the DTI group, the mean ranges for psychosocial well-being were 63% to 83.62%, and for sexual well-being, 49% to 73.86%. In the TE group, the corresponding ranges were 63% to 75.9% for psychosocial well-being, and 49% to 74.54% for sexual well-being. Breast satisfaction ranges were 65% to 80.24% for the DTI group and 65% to 75.65% for the TE group. An implant satisfaction score was only provided for 1 study (Salgarello et al, DTI: 63%) (Table 3).^39^

**Table 3.** BREAST-Q Scores in DTI and TE Reconstruction Groups

Study	Psychosocial well-being	Sexual well-being	Breast satisfaction	Implant satisfaction	Group
Salgarello et al^39^	68%	55%	57%	63%	DTI
Tedeschi et al^10^	83.62 ± 5.49	73.86 ± 9.98	80.24 ± 8.88	—	DTI
Baker et al^46^	63	49	65	—	DTI
Salgarello et al^47^	63.8 ± 3.2	63.4 ± 3.8	70.6 ± 5.0	—	DTI
Range (DTI)	63-83.62	49-73.86	65-80.24	—	DTI
Tedeschi et al^10^	72.85 ± 5.45	74.54 ± 8.44	75.65 ± 6.52	—	TE
Liu et al^44^	75.9 ± 19.3	51.6 ± 30.2	73.1 ± 22.0	—	TE
Baker et al^46^	63	49	65	—	TE
Range (TE)	63-75.9	49-74.54	65-75.65	—	TE

DTI, direct-to-implant; TE, tissue expander-to-implant.

Salgarello was the only study to distinguish BREAST-Q scores between subglandular and submuscular placement in DTI. However, the data from this single study were insufficient to calculate an average result, so we used the data from subglandular placement to determine the ranges for each domain of the score. The SDs of the score results were not provided for every study and were therefore not used in the determination of ranges.

## DISCUSSION

The authors of previous studies have compared outcomes between augmented and nonaugmented patients but, to our knowledge, this systematic review is the first to comprehensively assess all existing implant-based surgical techniques. Because of the limited number of studies and the paucity of available data, a statistical quantitative analysis was not feasible. Hence, we conducted a systematic review that highlights variability in complication rates between groups, with TE showing particularly higher rates, whereas patient satisfaction demonstrated more consistent ranges.

In our study, we found TE to be the most performed implant-based reconstruction, which correlates with ASPS data from 2023, followed by DTI.^37^ As previously highlighted, TE is typically performed in more complex cases or when radiation is expected. In our study, we found overall complication rates are higher in TE (54.8%) than in DTI (25.5%) and ISM (22.4%). Infection was the most prevalent complication in the TE group (14.8%). Specifically, Roostaeian et al and Sbitany et al indicated infection as more common among previously augmented patients, specifically naming cellulitis.^35,42^ Although timing of complications was inconsistently reported in these studies, in a review, a single surgeon found more complication occurrence during TE placement (8.5%) than during expander-to-implant exchange surgery (2.7%).^38,48^

The population samples in TE studies shared similar risk factors with the other groups, including age, BMI, hypertension, and diabetes. Diabetes rates were only higher in the study by Tedeschi et al; however, in that review, they did not show higher complication rates than other TE studies. In contrast, Liu et al did report more complication rates combined with results for augmentation and augmentation mastopexy, which may also explain in this study.

Another factor, which may explain the elevated complication rates in this group, is the need for multiple surgeries. Indeed, the TE group essentially is a 2-surgery group compared with the ISM and DTI groups; hence, naturally, the complication rates of infection would be higher—additional device exposure and possibly irradiation to the TE or implant.

The existence of residual biofilms from previous implants and less healthy tissue protecting the breast inevitably increases the infectious risk among these patients.^42^ The presence of bacteria or infection also seems to be linked to capsular contraction.^35,49-51^ However, we did not observe this tendency in our review, with capsular contracture being among the least frequent complications in TE and ISM groups. Although it is the most represented complication in the DTI group, its incidence remains low, at 5.11%. Based on the findings from this article, DTI would seem to be the best surgery for the least complications and highest patient satisfaction rates.

Necrosis was not a frequent complication in TE and DTI groups in this review. This observation concurs with multiple studies that also reported fewer incidences of necrosis among augmented patients. This is likely because of a “delay phenomenon,” in which previous alteration of perforators from the pectoralis and breast gland may help the nipple skin better tolerate reduced blood flow from mastectomy and the tension caused by expander/implant placement.^35,42^ In the ISM group, it was the most common complication, although it remained relatively low at 6.63%. However, some studies suggest that patients with a history of breast augmentation or mastopexy may be at an increased risk of ischemia and necrosis because of compromised vascularity.^10,52^

Although a total of 38 patients in our review underwent radiation therapy, and the rates were similar between the included studies, we did not have enough data to further analyze this factor. Nevertheless, in the case of TE during expander-to-implant surgery, it is important to consider the patient's history of radiation therapy. For these patients, surgeons are advised to minimize tension on the irradiated pectoralis muscle during the initial stages of reconstruction to reduce the risk of wound dehiscence, implant exposure, and other complications.^42^ For such patients, only a small volume of saline is typically injected at the time of expander placement, whereas patients without previous radiation may undergo more aggressive expansion to preserve skin flap form.^42^ If breast tissues are damaged because of previous radiation, or if postoperative radiation is expected, autologous reconstruction may be a more suitable option for patients, as any implant could be negatively affected.^45^

Our review did not study submuscular and subglandular placement as a variable because of a lack of data, but these options were mentioned in 3 studies.^10,35,39^ Roostaeian et al found no significant differences between previous subglandular and subpectoral implant subgroups in terms of postoperative complications, including capsular contracture.^35^ The lack of data on these implant planes in our review limits our ability to correlate complications with the choice of technique. Although we observed various complications, we could not determine whether they were associated with submuscular or subglandular placement, preventing us from extrapolating their impact on complication rates. Further investigation is needed to clarify their influence.

Submuscular placement in DTI or TE presented different issues associated with pectoralis muscle disruption (muscle retraction, implant malposition, and contracture).^53^ ADM was proposed as an effective remedy to these muscle-related complications, through better control of the implant pocket, when compared with the exclusive use of tissue expanders for example.^42,53^ However, ADM has since been related to increased complication rates as well.^39,54-56^ There are important discrepancies surrounding the safety of this product, highlighting the need for exploration and analysis at a larger scale. Although it was mentioned in some of the articles included in our review, we did not have enough data to study this variable between the different groups.

In our review, ADM was used in multiple studies from the DTI and TE groups.^10,35,39,42^ TE presented a 2% rate for necrosis and 2.4% for explantation. For the other groups, we found, respectively, 1.46% and 3.65% for DTI and 6.63% and 1.53% for ISM. These results between groups reflect the inconsistencies found in existing literature.

Among the included studies, we identified 4 cases of DTI reconstruction which were subsequently converted to autologous reconstruction. Additionally, 1 case of failed DTI reconstruction presented recurrent complications. The implant ultimately had to be removed in sight of the patient's extensive radiotherapy.

In our study, ISM has the lowest complication rate (22.4%) among the 3 methods. Furthermore, implant exchange in case of ISM is generally considered simpler and safer than expander placement surgery.^38^ In our review, the majority of patients from the studies by Burke et al, Le et al, and Prabhakaran et al did undergo exchange.^38,41,43^ The ISM technique functions as a form of temporary expansion and allows for better healing of the mastectomy skin flaps while awaiting definitive implant exchange, reducing the risk of skin complications in the initial recovery period.^38^ Necrosis was the most common complication in the ISM group but its overall incidence within the group remained low (6.63%). Additionally, Burke et al found a 9.9% overall unspecified return-to-OR rate for ISM, which is relatively similar to the 11.1% of the nonaugmented group.^43^

Prabhakaran et al compared ISM with TE reconstruction and found lower complication rates and similar tumor margins for ISM, despite similar smoking history and use of chemotherapy between groups, highlighting its safety and effectiveness.^38^ This aligns with the results of our review (22.4% complication rate for ISM vs 54.8% for TE). Moreover, patients who are already comfortable with breast implants are often more satisfied with the aesthetic outcomes provided by the ISM approach.^41^ The advantages of the technique in terms of reduced morbidity (shorter first operation time, delayed exchange, and fewer procedures compared with TE) make it an appealing option, particularly for patients seeking a more immediate aesthetically pleasing outcome.^34,38,41^ Careful patient selection and follow-up remain crucial to optimizing outcomes, but overall, ISM represents a viable option for many patients, with fewer complications and improved outcomes.

Although this strategy goes beyond the scope of our review, it is interesting to note that for patients with genetic mutations like breast cancer (BRCA) genes, preconstruction through breast augmentation followed by ISM is an alternative approach. This strategy offers the advantage of creating a breast capsule before the mastectomy, which can help stabilize the implant and potentially improve long-term outcomes.^41^

Regarding aesthetic outcomes and patient satisfaction, in our study, we found that psychosocial well-being and breast satisfaction scores were higher in DTI, whereas sexual well-being scores were most similar in both groups, although the ranges for both groups exhibit considerable overlap. According to the findings of the studies by Baker et al and Salgarello et al, they indeed suggest BREAST-Q scores for breast satisfaction, as well as physical well-being, are often higher in augmented patients pursuing DTI reconstruction than TE or nonaugmented patients. This reflects their better understanding of postoperative conditions, including anticipated appearance changes and physical limitations.^46,47^ In another study, Tedeschi et al found physical well-being scores among patients with previous submuscular augmentation to be higher than in those with subglandular placement, indicating that previous disruption of the muscle may attenuate the physical impact of subsequent subpectoral reconstruction.^10^ However, they found overall satisfaction scores to be superior in the subglandular reconstruction group. The sexual well-being domain had the lowest minimum score range, the same in both groups based on Baker et al (49%), which did not give individual BREAST-Q results per group.^46^

Liu et al exhibited a notably larger SD compared with the other reviews included for secondary outcomes, likely because of the relative higher number of breasts in this study (*n* = 111).^44^

This review has several limitations, notably the restricted number of studies available on the topic, with often small sample sizes and potential variability in study designs. This prevented us from conducting a meta-analysis, which may impact the generalizability of our results. The specificity of terminology used in our search strategy may have limited the number of identified articles. The 3 techniques included are fundamentally different in nature, requiring a different number of procedures, and have their own risk profiles. TE is a multistage approach that inherently presents higher risks, whereas DTI appears to be a favorable option with acceptable complication rates and good patient satisfaction. In our review, we aimed to provide an overview of associated complication rates; however, no direct comparison was possible. Ultimately, the choice of reconstruction should be guided by this information alongside various patient- and case-specific factors discussed in the review. Additionally, the included studies were primarily observational in nature and had variation in their outcome measures, such as differing follow-up durations, ranging from 15.3 months to 3 years, which could potentially bias complication rates. Finally, the lack of data did not allow for a multivariate analysis, which could have highlighted the potential role of risk factors, such as malnutrition, diabetes, and smoking in complication rates.

## CONCLUSIONS

The current literature describes 3 primary implant-based reconstructive techniques for previously augmented patients undergoing mastectomy, namely TE, ISM, and DTI. The most common approach is TE, followed by ISM and DTI. Patients requiring TE present a high number of complications, which appears to be less prevalent in ISM and DTI groups. Nonetheless, the BREAST-Q scores showed average-to-high patient satisfaction for patients in DTI and TE groups. However, further research with larger sample sizes and extended follow-up is essential to substantiate these findings.

## Supplementary Material

ojaf049_Supplementary_Data
