# Supplementary material for: Outcomes and Complications of Implant-Based Breast Reconstruction in Patients With Previous Cosmetic Augmentation: A Systematic Review
Source: Aesthet Surg J Open Forum. 2025 May 26;7:ojaf049. doi: 10.1093/asjof/ojaf049 (PMC12202881; doi:10.1093/asjof/ojaf049)
Supplement: ojaf049_Supplementary_Data [file ojaf049_supplementary_data.docx]

**Supplementary tables**

Supplemental Table 1: Research strategy

| Database | Date | Search query | N articles |
| --- | --- | --- | --- |
| PubMed | December 21st 2024 | Direct-to-Implant OR One-Stage Reconstruction OR Immediate Breast Reconstruction  OR Implant-Sparing OR Implant Preservation  OR Tissue expander OR two-Stage Reconstruction OR Secondary breast reconstruction  AND Breast Reconstruction OR “Mammaplasty"[MeSH Terms]  AND ("Breast Neoplasms"[MeSH Terms] OR "Mastectomy"[MeSH Terms])  AND Breast augmentation OR Augmented OR Augmentation | 816 |

Supplemental Table 2: Selection criteria according to PICOS

|  | Inclusion | Exclusion |
| --- | --- | --- |
| Population | Women with a history of previous cosmetic breast augmentation.  Women undergoing implant-based breast reconstruction or implant-sparing mastectomy. | Patients without previous cosmetic breast surgery |
| Intervention | Direct-to-implant reconstruction  Tissue expander-to-implant reconstruction  Implant-sparing mastectomy | Autologous reconstruction |
| Comparator | None  Between intervention groups |  |
| Outcomes | Primary: post-operative complications and return to the operating room rates  Secondary: patient satisfaction based on breast-q score | Studies not reporting the primary outcome |
| Study design | Prospective, retrospective, comparative | Case reports, case series (<5 cases), reviews. |
